# The Involvement of Mutual Inhibition of ERK and mTOR in PLCγ1-Mediated MMP-13 Expression in Human Osteoarthritis Chondrocytes

**DOI:** 10.3390/ijms160817857

**Published:** 2015-08-04

**Authors:** Zejun Liu, Heguo Cai, Xinpeng Zheng, Bing Zhang, Chun Xia

**Affiliations:** 1 Department of Sports Medicine & Joint Surgery, Zhongshan Hospital, Xiamen University, Xiamen 361004, China; E-Mails: 18850311056@163.com (Z.L.); caihg0911@163.com (H.C.); zxp_635@126.com (X.Z.); 2 The People’s Hospital, Hubei University of Medicine, Shiyan 442000, China; 3 Medical School, Xiamen University, Xiamen 361102, China

**Keywords:** ERK, PLCγ1, mTOR, MMP-13, OA chondrocytes

## Abstract

The issue of whether ERK activation determines matrix synthesis or degradation in osteoarthritis (OA) pathogenesis currently remains controversial. Our previous study shows that PLCγ1 and mTOR are involved in the matrix metabolism of OA cartilage. Investigating the interplays of PLCγ1, mTOR and ERK in matrix degradation of OA will facilitate future attempts to manipulate ERK in OA prevention and therapy. Here, cultured human normal chondrocytes and OA chondrocytes were treated with different inhibitors or transfected with expression vectors, respectively. The levels of ERK, p-ERK, PLCγ1, p-PLCγ1, mTOR, p-mTOR and MMP-13 were then evaluated by Western blotting analysis. The results manifested that the expression level of ERK in human OA chondrocytes was lower than that in human normal articular chondrocytes, and the up-regulation of ERK could promote matrix synthesis, including the decrease in MMP-13 level and the increase in Aggrecan level in human OA chondrocytes. Furthermore, the PLCγ1/ERK axis and a mutual inhibition of mTOR and ERK were observed in human OA chondrocytes. Interestingly, activated ERK had no inhibitory effect on MMP-13 expression in PLCγ1-transformed OA chondrocytes. Combined with our previous study, the non-effective state of ERK activation by PLCγ1 on MMP-13 may be partly attributed to the inhibition of the PLCγ1/mTOR axis on the PLCγ1/ERK axis. Therefore, the study indicates that the mutual inhibition of ERK and mTOR is involved in PLCγ1-mediated MMP-13 expression in human OA chondrocytes, with important implication for the understanding of OA pathogenesis as well as for its prevention and therapy.

## 1. Introduction

Osteoarthritis (OA) is a chronic degenerative joint disease, resulting in severe pain and physical disabilities for millions of people worldwide. The degradation of cartilage matrix is one of OA’s pathological features. Chondrocytes, which are responsible for the homeostatic maintenance of cartilage matrix, produce a variety of matrix-anabolic/catabolic enzymes or inhibitors, including matrix metalloproteinases (MMPs), aggrecanases and tissue inhibitor of metalloproteinases (TIMPs) [1]. Multiple signal molecules are involved in the regulation of cartilage matrix synthesis, and elucidating their regulatory mechanisms in chondrocytes is beneficial to preventing cartilage damage and promoting repair.

Extracellular signal-regulated kinase (ERK) is one of the MAPK families that is activated by a variety of environmental stress and inflammatory cytokines. Activated ERK mediates cellular responses to intracellular signaling proteins and is involved in a number of physiological and disease processes, including RA (Rheumatoid Arthritis) and OA. As an example, the activation of DDR-2 (discoidin domain receptor 2) by intact type II collagen fibrils leads to increased MMP-13 expression via the Ras/Raf/MEK/ERK pathway and p38 signaling in hereditary OA [2]. MEK/ERK signaling enhances ELF3 (E74-like factor 3)-driven MMP-13 transactivation and is required for IL-1β-induced ELF3 binding to the MMP13 promoter in articular chondrocytes under pro-inflammatory stress [3]. These studies seem to support the view that the activation of ERK induced by extracellular factors facilitates MMP-13 expression in OA chondrocytes. However, recent studies showed a beneficial role of ERK in matrix anabolism of chondrocyte. Progranulin activates ERK signaling and elevates the levels of anabolic biomarkers in degenerative human chondrocyte [4]. Bovine lactoferricin induces TIMP-3 via the ERK-Sp1 axis in human articular chondrocytes [5]. Therefore, the issue of whether ERK activation determines matrix synthesis or degradation in OA pathogenesis currently remains controversial.

Multiple signaling pathways always are triggered by common upstream stimuli and coordinately regulated by extensive crosstalk. Studying signal pathways individually or ignoring potential pathway interplays that can result in positive or negative effects of one pathway on another leads to the mutual antagonism between inhibitors. A better understanding of the regulatory mechanism of major signal pathways in OA chondrocytes will facilitate future attempts to manipulate them in OA prevention and therapy. The mechanistic target of rapamycin (mTOR) pathway is a serine-threonine protein kinase, and has been found to be crucial in controlling protein synthesis [6]. Recent study has shown that cartilage-specific deletion of mTOR protects mice from OA [7]. Furthermore, the pharmacological inhibition of mTOR can lead to the up-regulation of MAPK through the p70S6K-PI3K-Ras signaling cascade modulated by IRS-1 in human cancer [8], indicating the possibility of the existence of the interplay of mTOR and ERK in OA chondrocytes. Furthermore, phosphoinositide-specific phospholipases γ (PLCγ), as one of the phosphoinositide-specific phospholipases families [9,10], has recently been shown to be involved in the sex-specific response of rat costochondral cartilage growth plate chondrocytes to 17β-estradiol [11]. Especially, our previous study showed that PLCγ1 has higher expression and promotes matrix degradation by triggering the mTOR pathway in human OA chondrocytes [12]. These studies display the involvement of PLCγ1 in chondrocyte metabolism and the existence of PLCγ1/mTOR axis. In addition, the association between Ras-GRF1/2 and PLCγ1 is required for Ras signaling, ERK activation and MMP-3 release downstream of IL-1 stimulation in NIH 3T3 fibroblasts, showing the crosstalk between ERK and PLCγ1 [13]. Therefore, it is confirmed that the interplays of PLCγ and mTOR or PLCγ and ERK are involved in cell metabolism in some cell types [8,12,13]. Otherwise, the crosstalk of PLCγ1, mTOR, and ERK in OA pathogenesis is not elucidated.

In this study, we investigated the interplays of PLCγ1, mTOR, and ERK in human OA chondrocytes. It is determined that the mutual inhibition of ERK and mTOR is involved in PLCγ1-mediated MMP-13 expression in human OA chondrocytes, which is beneficial to understanding the pathogenic mechanism of OA as well as for its prevention and therapy.

## 2. Results

### 2.1. The Effect of ERK on Matrix Synthesis in Human OA Chondrocytes

**Figure 1:**
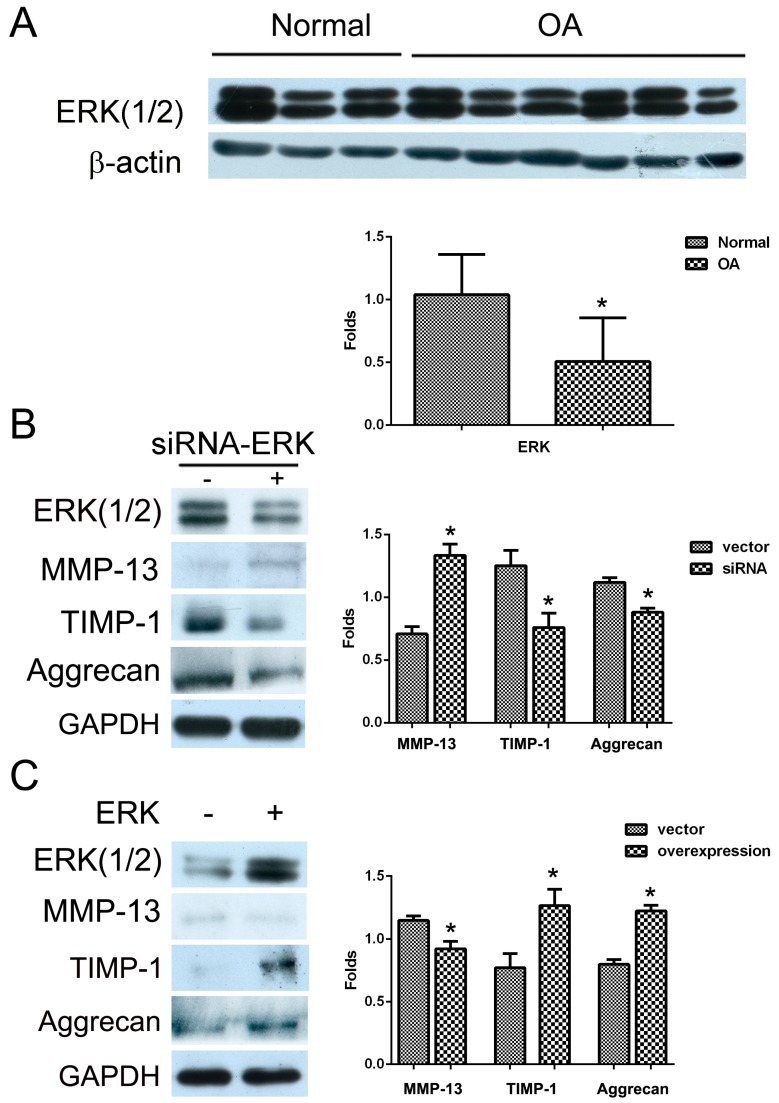
The effect of ERK on matrix synthesis in human OA chondrocytes. (**A**) Normal and OA chondrocytes were cultured, and the level of ERK(1/2) was detected by Western blotting analysis using anti-ERK(1/2) and β-actin antibodies; (**B**) Cells were transfected with siRNA/ERK vector for seven days and the levels of MMP-13, TIMP-1 and Aggrecan were detected by Western blotting analysis using anti-MMP-13, TIMP-1, Aggrecan and GAPDH antibodies; (**C**) Cells were transfected with ERK vector for two days and the levels of MMP-13, TIMP-1 and Aggrecan were detected by western blotting analysis using anti-MMP-13, TIMP-1, Aggrecan and GAPDH antibodies. The blots were normalized to an endogenous protein (β-actin or GAPDH). The values represent the mean ± SEM of three or five independent experiments (patients), each yielding similar results (* *p* < 0.05).

Human normal and OA chondrocytes obtained from patients were cultured, respectively, and the expression levels of ERK(1/2) were measured with Western blotting analysis. The results of Figure 1A showed that the level of ERK(1/2) in human OA chondrocytes was lower than that in normal human articular chondrocytes (Figure 1A, * *p* < 0.05). Furthermore, the depletion of ERK by siRNA led to the increase in the level of MMP-13 and the decrease in the levels of TIMP-1 and Aggrecan (Figure 1B, * *p* < 0.05). In contrast, the transfection of ERK vector in human OA chondrocytes led to the decrease in the level of MMP-13, while the levels of TIMP-1 and Aggrecan were up-regulated (Figure 1C, * *p* < 0.05).Therefore, ERK could promote matrix synthesis in human OA chondrocytes.

### 2.2. The Effect of PLCγ1 on the Activation of ERK in Human OA Chondrocytes

**Figure 2:**
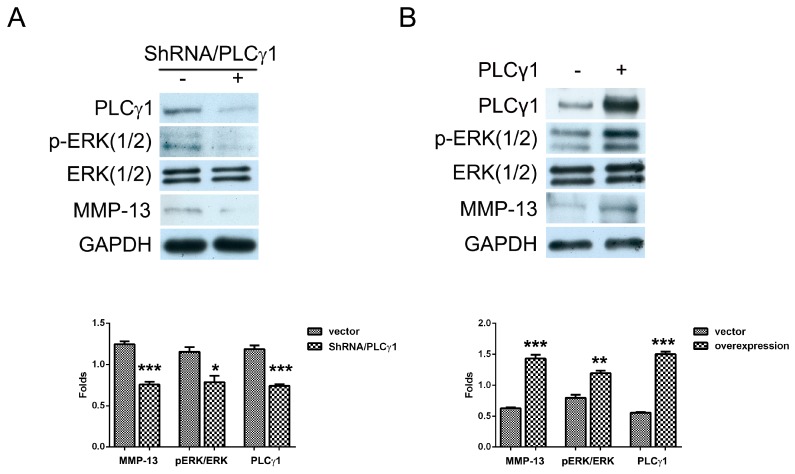
The effect of PLCγ1 on the activation of ERK in human OA chondrocytes. (**A**) Cells were transfected with ShRNA/PLCγ1 vector for seven days, and the levels of ERK(1/2), p-ERK(1/2) and MMP-13 were detected by Western blotting analysis using anti-ERK(1/2), p-ERK(1/2), MMP-13 and GAPDH antibodies; (**B**) Cells were transfected with PLCγ1 vector for two days and the levels of ERK(1/2), p-ERK(1/2) and MMP-13 were detected by western blotting analysis using anti-ERK(1/2), p-ERK(1/2), MMP-13, and GAPDH antibodies. The blots were normalized to an endogenous protein (GAPDH). The values represent the mean ± SEM of three or five independent experiments (patients), each yielding similar results (* *p* <0.05, ** *p* <0.01, *** *p* < 0.001).

To determine the interaction of PLCγ1 and ERK in OA chondrocytes, human OA chondrocytes cultured were transfected with ShRNA-PLCγ1 and PLCγ1 vectors, respectively. The depletion of PLCγ1 by ShRNA led to the decrease in the level of p-ERK(1/2) (Figure 2A, * *p* < 0.05). In contrast, the transfection of PLCγ1 vector led to the increase of p-ERK(1/2) level, in which that of p44 (ERK1) was more than that of p42 (ERK2) (Figure 2B,** *p* < 0.01). However, activated ERK by PLCγ1 did not inhibit MMP-13 expression in PLCγ1-transformed OA chondrocytes, while MMP-13 expression increased in PLCγ1-transformed OA chondrocytes (Figure 2A,B, *** *p* < 0.001). Therefore, the tendency of MMP-13 expression coincided with that of PLCγ1 expression, not ERK expression, implying that the activation of ERK by PLCγ1 had no inhibitory effect on MMP-13 expression.

### 2.3. Mutual Inhibition of ERK and mTOR Signaling in Human OA Chondrocytes

**Figure 3:**
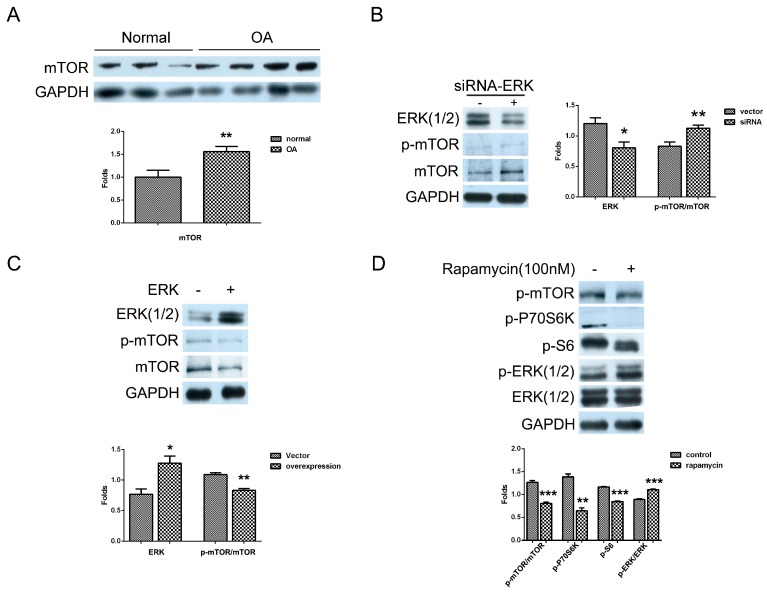
The mutual inhibition of mTOR and ERK in human OA chondrocytes. (**A**) Normal and OA chondrocytes were cultured, and the level of mTOR was detected by western blotting analysis using anti-mTOR, p-mTOR, and GAPDH antibodies; (**B**) Cells were transfected with siRNA/ERK vector for seven days and the levels of mTOR and p-mTOR were detected by Western blotting analysis using anti-mTOR, p-mTOR and GAPDH antibodies; (**C**) Cells were transfected with ERK vector for two days and the levels of mTOR and p-mTOR were measured with western blotting analysis; (**D**) Cells were treated with rapamycin (100 nM) for 2 h and the levels of p-mTOR, p-P70S6K, p-S6, ERK(1/2), and p-ERK(1/2) were detected by western blotting analysis using anti-mTOR, p-mTOR, p-P70S6K, p-S6, ERK(1/2), p-ERK(1/2) and GAPDH antibodies. The blots were normalized to an endogenous protein (GAPDH). The values represent the mean ± SEM of three independent experiments (patients), each yielding similar results (* *p* < 0.05, ** *p* < 0.01, *** *p* < 0.001).

A previous study showed that PLCγ1 reduced the extracellular matrix synthesis by triggering the mTOR/P70S6K/S6 pathway in human OA chondrocytes [12]. Here, we detected the level of mTOR in human normal and OA chondrocytes with Western blotting analysis. Compared to normal chondrocytes, mTOR has higher expression in OA chondrocytes (Figure 3A, ** *p* < 0.01). Furthermore, to investigate the relationship between mTOR and ERK, human OA chondrocytes cultured were transfected with siRNA-ERK and ERK vectors, respectively, and the levels of mTOR and p-mTOR were detected with western blotting analysis. The depletion of ERK by siRNA led to the increase in mTOR and p-mTOR level (Figure 3B, ** *p* < 0.01) and the transfection with ERK vector led to the decrease in mTOR and p-mTOR level (Figure 3C, ** *p* < 0.01). Afterwards, the addition of mTOR inhibitor, rapamycin (100 nM), led to the increase in p-ERK(1/2) level, in which the effect of rapamycin on p44 (ERK1) and p42 (ERK2) seemed to be different (Figure 3D). Meanwhile, rapamycin suppressed the mTOR/P70S6K/S6 pathway (Figure 3D, ** *p* < 0.01, *** *p* < 0.001). These results indicated the existence of a mutual inhibition of mTOR and ERK signaling in human OA chondrocytes.

### 2.4. The PLCγ1/mTOR Axis Was a Dominating Pathway in Human OA Chondrocytes, Compared to the PLCγ1/ERK Axis

**Figure 4:**
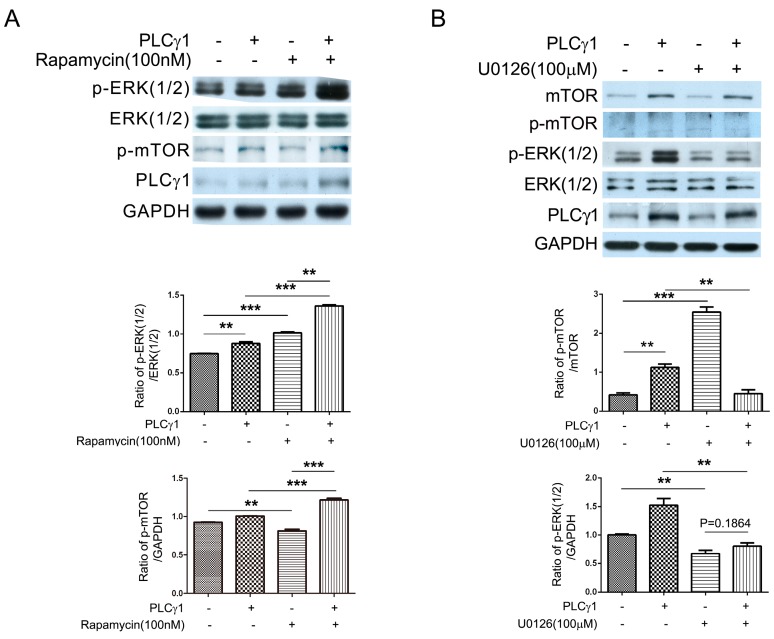
The mutual inhibition of mTOR and ERK in PLCγ1-transformed human OA chondrocytes. (**A**) Cells were transfected with PLCγ1 vector for two days followed by the treatment of rapamycin (100 nM) for 2 h, and the levels of ERK(1/2), p-ERK(1/2), p-mTOR, and PLCγ1 were detected by western blotting analysis using anti-ERK(1/2), p-ERK(1/2), p-mTOR, PLCγ1 and GAPDH antibodies; (**B**) Cells were transfected with PLCγ1 vector for two days followed by the treatment of U0126 (100 μM) for 2 h and the levels of mTOR, p-mTOR, ERK(1/2), p-ERK(1/2) and PLCγ1 were detected by Western blotting analysis using anti-mTOR, p-mTOR, ERK(1/2), p-ERK(1/2), PLCγ1 and GAPDH antibodies. The blots were normalized to an endogenous protein (GAPDH). The values represent the mean ± SEM of three or five independent experiments (patients), each yielding similar results (** *p* <0.01, *** *p* < 0.001).

To investigate the mutual inhibition of mTOR and ERK in PLCγ1-transformed human OA chondrocytes, cells were transfected with PLCγ1 vector prior to the treatment with rapamycin (mTOR inhibitor) or U0126 (MEK/ERK inhibitor). The transfection with PLCγ1 vector led to the increase in p-ERK(1/2) level the same as the data of Figure 2B (Figure 4A, ** *p* < 0.01). The addition of rapamycin (100 nM) promoted the increase in p-ERK(1/2) level in human OA chondrocytes with or without PLCγ1 vector (Figure 4A, *** *p* < 0.001). The transfection with PLCγ1 vector enhanced the promoting effect of rapamycin (100 nM) on p-ERK level (Figure 4A, ** *p* < 0.01). Interestingly, the tranfection with PLCγ1 vector offset the inhibitory effect of 100 nM rapamycin on p-mTOR in OA chondrocytes (Figure 4A, *** *p* < 0.001), in spite of that the same concentration of rapamycin (100 nM) could inhibit the level of p-mTOR in OA chondrocytes transfected with empty vector (Figure 4A, ** *p* < 0.01). Meanwhile, the addition of ERK inhibitor U0126 (100 μM) did not lead to the increase in p-mTOR level in PLCγ1-transformed OA chondrocytes (Figure 4B, ** *p* < 0.01), even if the same concentration of U0126 (100 μM) could elevate the level of p-mTOR in OA chondrocytes with empty vector (Figure 4B, *** *p* < 0.001), indicating that the transfection with PLCγ1 vector attenuated the promoting effect of U0126 on the level of p-mTOR. The transfection with PLCγ1 vector did not significantly attenuate the inhibitory effect of U0126 on the level of p-ERK (Figure 4B, *p* > 0.05). The data thus manifested that the promoting effect of mTOR inhibitor, rapamycin, on the level of p-ERK was stronger than that of MEK/ERK inhibitor, U0126, on the level of p-mTOR in PLCγ1-transformed OA chondrocytes. Combined with the higher expression of PLCγ1 and mTOR in human OA chondrocytes (Figure 3A) [12], it is implied that the PLCγ1/mTOR axis was a dominating axis in human OA chondrocytes, compared to the PLCγ1/ERK axis.

## 3. Discussion

In this study, it is demonstrated that ERK has lower expression in human OA chondrocytes compared to normal articular chondrocytes and the up-regulation of ERK promoted matrix synthesis in human OA chondrocytes, including the decrease of MMP-13 level and the increase of Aggrecan. Meanwhile, our data indicates the existence of the PLCγ1/ERK axis in human OA chondrocytes. However, the activation of ERK by PLCγ1 had no inhibitory effect on MMP-13 expression, which may be attributed to the inhibition of the PLCγ1/mTOR axis on the PLCγ1/ERK axis in human OA chondrocytes. Therefore, the mutual inhibition of ERK and mTOR is involved in PLCγ1-mediated up-regulated MMP-13 expression in human OA chondrocytes, suggesting the existence of crosstalk among PLCγ1, mTOR and ERK in OA pathogenesis. It is beneficial to understanding the regulatory mechanism of ERK in OA pathological progression.

Fan *et al.* [14] reported that active ERK signal is detected in lysates of freshly-isolated articular chondrocytes and all normal and OA tissue samples. We also measured the expression of ERK in cultured human normal and OA chondrocytes. Thus, it is confirmed that ERK is expressed in both normal and OA articular chondrocytes. Meanwhile, we demonstrated that the total level of ERK declined in OA chondrocytes obtained from six patients compared to normal chondrocytes obtained from three donors. This is in agreement with the results in Fan *et al.* [14] data despite that it was not described. Furthermore, the up-regulation of ERK promoted matrix synthesis in OA chondrocytes, consistent with other authors’ studies, such that activated ERK signaling by pro-granulin elevated the levels of anabolic biomarkers in human chondrocyte [4], also there is our recent study that the activation of ERK by morroniside promotes matrix synthesis in human OA chondrocytes [15]. Based on these studies, it is inferred that ERK is a beneficial role in OA therapy. However, some studies state that ERK exhibits a disruptive effect on matrix synthesis in OA chondrocyte and extracellular factors prevent cartilage degradation of OA through inhibiting the activation of ERK [2,16,17,18]. From our own perspective, the contradictory role of ERK may be associated with the different site of obtained chondrocytes in those studies, including the superficial, intermediate and deep layers of cartilage. Chondrocytes in different layers of cartilage own their respective providers of nutrients, and are exposed to different regulatory factors, resulting the fact that the same signaling molecule expresses different level and exhibits different effects in OA pathogenesis [19,20]. The study of Fan *et al.* has shown the differential expression of ERK in OA cartilage layers, implying that ERK in different layers of cartilage could play different roles in OA pathogenesis [14]. Therefore, considering the distribution of ERK in cartilage is required for understanding the role of ERK in OA therapy.

In addition, ERK1(p44) and ERK2(p42) have been reported to exert different effect on regulating cell metabolism. As an example, ERK2 but not ERK1 regulates the crosstalk between Met and EGFR in squamous cell carcinoma cell lines [21]. Active ERK2 is sufficient to mediate growth arrest and differentiation signaling [22]. In the present, the different expression of ERK1 and ERK2 in PLCγ1-transformed chondrocytes was also observed, implying that ERK1 and ERK2 could play different roles in regulating MMP-13 expression in PLCγ1-transformed chondrocytes. The differently regulatory mechanism between ERK1 and ERK2 needs to be studied further.

As one of the important signal molecules, ERK has been known to be involved in multiple signal pathways. Recent study showed that the Src-PLCγ1-ERK1/2 signaling transduction pathway is involved in cartilage tissue integration by affecting chondrocyte migration [23]. Here, our findings also indicated the existence of the PLCγ1/ERK axis in OA chondrocytes. However, the activated ERK by PLCγ1 did not exert its promoting effect on matrix synthesis in OA chondrocytes, implying that the PLCγ1/ERK axis was blocked by other signal axes. Furthermore, our data indicates the existence of a mutual inhibition of mTOR and ERK in human OA chondrocytes, in agreement with the functional crosstalk between Akt/mTOR and Ras/MAPK pathways in hepatocarcinogenesis [24]. Therefore, the non-effective inhibitory effect of PLCγ1/ERK axis on MMP-13 expression could be attributed to the mutual inhibition of mTOR and ERK. It has been recently reported that the inhibition of the Akt/mTOR pathway resulted in increased activation of ERK1/2 signaling in differentiating oligodendrocytes, but not vice versa [25]. In the present, the inhibitor of mTOR also exerted stronger effect on ERK level than that of ERK inhibitor on mTOR level in PLCγ1-transformed OA chondrocytes, indicating that the inhibition of mTOR pathway significantly increased ERK signaling; and the inhibition of the ERK pathway does not have enough impact on mTOR signaling, consistent with the very recent study in neuropathic pain [26]. Combined with the data of the existence of higher expressed the PLCγ1 and mTOR, and lower expressed ERK in human OA chondrocytes, we suggest that PLCγ1/ERK axis was blocked by the PLCγ1/mTOR axis, and the latter inhibited the effect of the PLCγ1/ERK axis on MMP-13 expression in human OA chondrocytes.

## 4. Experimental Section

### 4.1. Reagents and Antibodies

Antibodies against PLCγ1 (CST#2822S), p-PLCγ1-Tyr783 (CST#2821S), mTOR (CST#2983), p-mTOR-Ser2448 (CST#2983), ERK1/2 (CST#4695), p-ERK1/2-Thr202/Tyr204 (CST#9106), siRNA-ERK (CST#6560), TIMP-1 (tissue inhibitor of metalloproteinase-1, CST#8946), and GAPDH (CST#2251-1) were purchased from Cell Signaling Technology Inc. (Beverly, MA, USA). Antibodies against MMP-13 (SC-30073), AGG (SC-16493), and Col II (SC-52658) were obtained from Sigma-Aldrich in China (Shanghai, China). Other reagents were of the highest grade commercially available.

### 4.2. Human Normal and OA Chondrocyte Isolation and Culture

Ethical approval for the study was obtained from the Ethics Committee of Zhongshan Hospital, Xiamen University (ID No. 20100426), China. After receiving all patient consent and in accordance with the hospital ethical guidelines, human normal cartilage was obtained from 4 patients (aged 22–45 years, 4 males) with amputation from an accident and OA cartilage was obtained from 50 patients (aged 60–76 years, 7 males and 43 females) with advanced OA who were undergoing total knee replacement surgery. In order to avoid the selection bias, these patients were selected randomly (Table 1 and Supplementary Figure S1A). The OA patients were diagnosed based on the criteria developed by the American College of Rheumatology Diagnostic Subcommittee for OA and had not taken any non-steroidal anti-inflammatory drugs or steroids for at least two weeks prior to surgery or had any intra-articular injection for at least 1 month prior to surgery. Articular cartilage was dissected from the femoral condyle and tibial plateau; one part was stored in liquid nitrogen for chondrocyte culture; another part was fixed in 4% paraformaldehyde for immunohistochemistry technique.

As described previously [12,27], the cartilage slices were minced and digested primarily with collagenase II for overnight in serum-free DMEM/F12 at 37 °C, collected by centrifugation (1000× *g* for 5 min), re-suspended and cultured in DMEM/F12 supplemented with 10% FBS (*v*/*v*) plus 1% penicillin/streptomycin. Prior to being used in the experiments, the expression of Col II (the main collagen type in cartilage matrix), in OA chondrocytes was detected with the immunohistochemistry technique in order to manifest the characteristics of chondrocytes (Supplementary Figure S1B). All of our clinical studies have been conducted according to the principles expressed in the Declaration of Helsinki.

**Table 1:** Information of osteoarthritis (OA) patients with total knee replacement surgery.

Age (year)	Case	Sex		Duration of OA (year)		* K.L. Image Criterion				Pro-Treatment
		Male	Female	≤3	>3	I	II	III	IV	
60**-**	23	1	22	7	16	0	2	12	9	0
65**-**	27	6	21	7	20	1	3	10	13	0

* K.L Image criterion: Kellgren and Lawrecne criterion.

### 4.3. Plasmid Construction and Transient Transfection

Rat PLCγ1 cDNA was N-terminally tagged with the HA sequence and subcloned into pRK5-HA (pRK5-HA/PLCg1). The vector of expressing PLCγ1 ShRNA and empty vector were purchased from Shanghai Genechem Co., Ltd. (Shanghai, China). The target sequence of PLCγ1 ShRNA is AGGGAAACAAAGTTTACAT. The vector of expressing ERK was presented by Dr. Jiahuai Han (Xiamen University). SiRNA-ERK was obtained from Sigma-Aldrich in China (Shanghai, China). The different expression vectors of PLCγ1 and ERK were transfected into cells using Lipofectamine 2000 according to the manufacturer’s procedure (Invitrogen, Carlsbad, CA, USA), after human OA chondrocytes were cultured in a 60-mm dish for 20–24 h until they reached 60%–70% confluence.

### 4.4. Protein Extraction and Western Blotting Analysis

Cells collected by centrifugation were lysed as previously described [28]. Protein extracts were electrophoresed on 8%–12% denaturing gel and transferred to PVDF membrane (GE Healthcare, Hertfordshire, UK) for Western blotting analysis [28]. The signal was detected using a chemiluminescent detection system according to the manufacturer’s instructions (Pierce, Rockford, IL, USA).

### 4.5. Statistical Analysis

The differences between the groups were examined for statistical significance using Student’s *t*-test with GraphPad Prism 5 software (GraphPad Software, La Jolla, CA, USA). A value of *p* < 0.05 was considered as being significant.

## 5. Conclusions

**Scheme 1:**
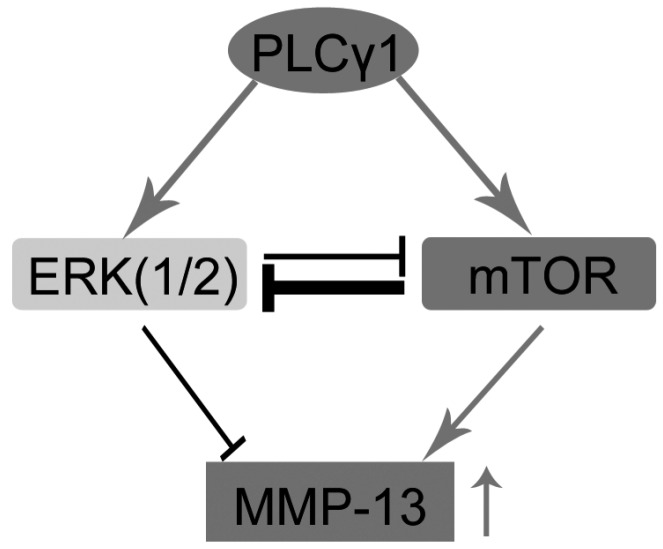
Schematic representation of mutual inhibition between ERK(1/2) and mTOR in PLCγ1-mediated up-regulated MMP-13 expression in human OA chondrocytes.

In conclusion, the up-regulation of ERK could promote matrix synthesis. There is the existence of the PLCγ1/ERK axis and mutual inhibition of mTOR and ERK in OA chondrocytes. ERK did not exert its promoting effect on matrix synthesis, in part, associated with the inhibition of the PLCγ1/mTOR axis on PLCγ1/ERK axis in human OA chondrocytes. Therefore, ERK could play an opposing role in the regulation of PLCγ1 on matrix degradation of human OA chondrocytes (Scheme 1).

## Supplementary Materials

Supplementary materials can be found at http://www.mdpi.com/1422-0067/16/08/17857/s1.
