# Supplementary material for: The Involvement of Mutual Inhibition of ERK and mTOR in PLCγ1-Mediated MMP-13 Expression in Human Osteoarthritis Chondrocytes
Source: Int J Mol Sci. 2015 Aug 4;16(8):17857–69. doi: 10.3390/ijms160817857 (PMC4576213; doi:10.3390/ijms160817857)
Supplement: Supplementary file 1 [file ijms-16-17857-s001.pdf]

Supplementary Information

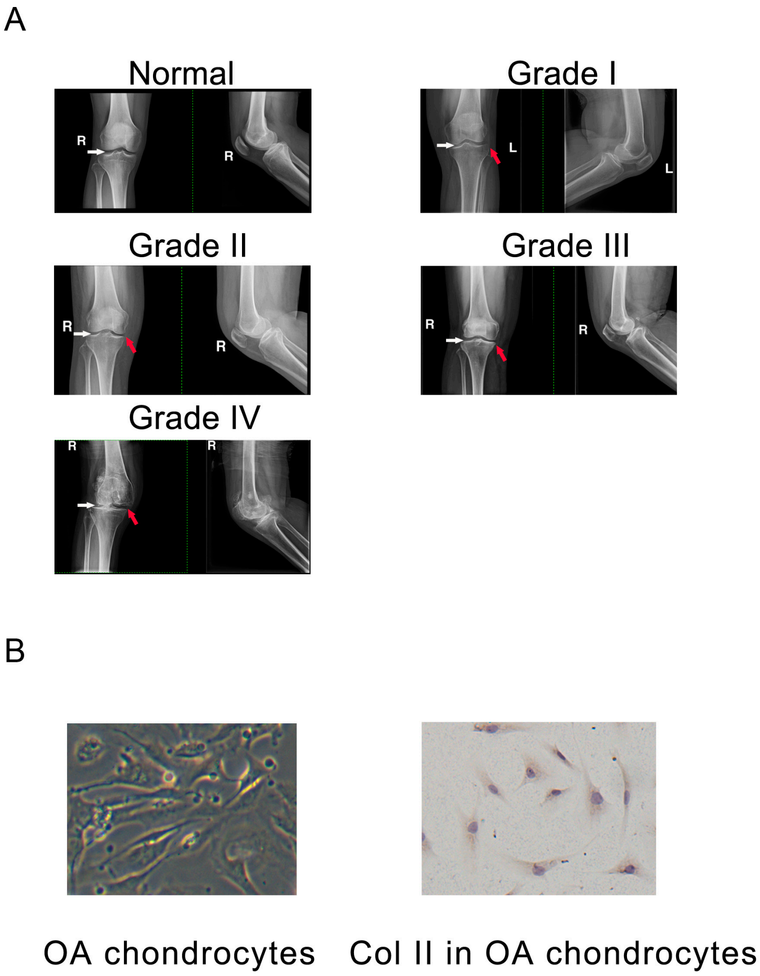

Figure S1. (**A**) The four grades of OA patients undergoing total knee replacement surgery according to K.L. Image Criterion (the white arrow indicates joint space and the red arrow indicates osteophyte spur); (**B**) The expression of collagen II in human OA chondrocytes using immunohistochemistry assay (magnification 100×).
